# Characteristics and neighborhood-level opportunity of assault-injured children in Milwaukee

**DOI:** 10.1186/s40621-023-00453-6

**Published:** 2023-08-21

**Authors:** Christina Georgeades, Manzur Farazi, Carisa Bergner, Alexis Bowder, Laura Cassidy, Michael N. Levas, Mark Nimmer, Katherine T. Flynn-O’Brien

**Affiliations:** 1 Division of Pediatric Surgery, Department of Surgery, Medical College of Wisconsin, Children’s Corporate Center, Suite C320, 999 N 92nd St, Milwaukee, WI 53226 USA; 2 Department of Epidemiology and Social Sciences, Medical College of Wisconsin, Milwaukee, WI USA; 3 Division of Pediatric Emergency Medicine, Department of Pediatrics, Medical College of Wisconsin, Milwaukee, WI USA; 4 Department of Pediatrics, Medical College of Wisconsin, Milwaukee, WI USA

**Keywords:** Pediatric trauma, Violent injury, Violent trauma, Assault-related injuries, Intentional injuries, Trauma reinjury

## Abstract

**Background:**

Multiple studies have explored demographic characteristics and social determinants of health in relation to the risk of pediatric assault-related injuries and reinjury. However, few have explored protective factors. The Child Opportunity Index (COI) uses neighborhood-level indicators to measure ‘opportunity’ based on factors such as education, social environment, and economic resources. We hypothesized that higher ‘opportunity’ would be associated with less risk of reinjury in assault-injured youth.

**Methods:**

This was a single-institution, retrospective study at a Level 1 Pediatric Trauma Center. Trauma registry and electronic medical record data were queried for children ≤ 18 years old with assault-related injuries from 1/1/2016 to 5/31/2021. Reinjured children, defined as any child who sustained more than one assault injury, were compared to non-reinjured children. Area Deprivation Index (ADI), a marker of socioeconomic status, and COI were determined through census block and tract data, respectively. A post-hoc analysis examined COI between all assault-injured children, unintentionally injured children, and a state-based normative cohort representative of non-injured children.

**Results:**

There were 55,862 traumatic injury encounters during the study period. Of those, 1224 (2.3%) assault injured children were identified, with 52 (4.2%) reinjured children and 1172 (95.8%) non-reinjured children. Reinjured children were significantly more likely to be older (median age 15.0 [IQR 13.8–17.0] vs. median age 14.0 [IQR 8.8–16.0], p < 0.001) and female (55.8% vs. 37.5%, p = 0.01) than non-reinjured children. COI was not associated with reinjury. There were also no significant differences in race, ethnicity, insurance status, ADI, or mechanism and severity of injury between cohorts. Post-hoc analysis revealed that assault-injured children were more likely to live in areas of lower COI than the other cohorts.

**Conclusions:**

Compared to children who sustained only one assault during the study period, children who experienced more than one assault were more likely to be older and female. Furthermore, living in an area with more or less opportunity did not influence the risk of reinjury. However, all assault-injured children were more likely to live in areas of lower COI compared to unintentionally injured and a state-based normative cohort. Identification of factors on a social or environmental level that leads to assaultive injury warrants further exploration.

**Supplementary Information:**

The online version contains supplementary material available at 10.1186/s40621-023-00453-6.

## Background

In 2020, assault injuries were the second leading cause of death in children (Centers for Disease Control and Prevention 2020). Assault injuries, which can include firearm injuries, stab wounds, burns, physical assaults, and child abuse, result in significant morbidity and mortality in addition to unique psychosocial needs (Academy and of Pediatrics Task Force on Adolescent Assault Victim Needs 1996; Cunningham et al. 2015; Zun and Rosen 2003). Of children that experience an assault injury, there are some that are at risk of reinjury, which is a repeat presentation for a separate traumatic assault-related injury. Potentially due to variation in how studies measure reinjury, the rate of pediatric reinjury varies greatly, with studies reporting rates of 1%-37% (Cunningham et al. 2015; Chong et al. 2015; Cortolillo et al. 2020; Gibson et al. 2016; Tellez et al. 1995).

A few studies have explored demographic characteristics, socioeconomic factors, and neighborhood disadvantage associated with assault injury and subsequent reinjury, finding that older age, male sex, Black race, lower socioeconomic status, and greater neighborhood deprivation were risk factors (Cunningham et al. 2015; Chong et al. 2015; Gibson et al. 2016; Carter et al. 2017). However, there is still a paucity of literature regarding these topics. Additionally, no published studies have explored protective factors and their effect on the risk of reinjury in children. The Child Opportunity Index (COI) utilizes neighborhood-level indicators to measure and map the degree of opportunity and protective factors a child has based on quality of resources such as education, health, social environment, and economic resources (Noelke et al. 2020). Existence of such protective factors, or the lack of them, could uniquely influence the risk of assault reinjury.

The objective of this study was to explore COI as a protective factor against reinjury among assault-injured youth in addition to demographic and injury characteristics that may be risk factors for reinjury. We also explored the impact of COI on assault-injured and non-assault injured cohorts. We hypothesized that 1) reinjured children would be more likely to live in areas of lower COI, and that 2) reinjured children would have significantly different demographic and injury characteristics than non-reinjured children.

## Methods

### Study population

A retrospective study of children ≤ 18 years old with assault-related injuries was performed at a single-institution Level 1 Pediatric Trauma Center from 1/1/16 to 5/31/2021. The institution where the study was performed is one of the main referral centers for a large part of the state and treats the majority of assault-injured youth that are moderately or severely injured. The Trauma Registry (TR) was queried in addition to the electronic medical record (EMR) to capture children treated and released from the Emergency Department who did not qualify for the TR. All traumatic injury encounters during the study period were identified for inclusion in the study. Subsequently, children with assault injuries were defined as having at least one assault injury, as identified by International Classification of Diseases, 10th revision (ICD-10) diagnosis codes (Additional file 1). Children with self-inflicted injuries were excluded.

Reinjury was defined as any child who sustained more than one assault injury within the study period. Children with reinjury were identified by a second encounter in the study period with an assault-related injury ICD-10 diagnosis code. There was no specific time period or limitation set regarding follow-up for reinjury for each child; reinjury could have occurred at any point within the study period. The “Reinjured cohort” was compared to the “Non-Reinjured cohort,” which included children that experienced no more than one assault-related injury. This study was reviewed and approved by the Children’s Wisconsin Institutional Review Board.

### Demographic and injury characteristics

Demographic information included age, sex, race, ethnicity, and insurance status. Age was identified as age at index injury for both the reinjured and non-reinjured cohort. Injury characteristics included mechanism of injury and the Injury Severity Score (ISS), which were determined from ICD-10 codes. ISS is captured by the TR through trained Abbreviated Injury Scale/ISS coders. Because ISS is not captured by the EMR system, ICDPIC-R was used to map ICD-10 diagnosis codes to ISS (Clark et al. 2018; The Comprehensive R Archive Network 2022). This was completed for children in both the TR and EMR for consistency. The calculated ISS was compared to the TR-based ISS through the concordance correlation coefficient to test the reliability of the calculated ISS (R Documentation 2022).

### Geographic and socioeconomic measures

Census blocks groups for the residential addresses of children were used to identify Federal Information Processing Standard codes to determine the Area Deprivation Index (ADI), which is a neighborhood-level measure of socioeconomic status. ADI ranks socioeconomic status through 17 factors which include employment, education level, housing quality, and income. ADI is reported as deciles 1 through 10, with decile 1 and 10 representing the least and most disadvantaged neighborhoods, respectively (Kind and Buckingham 2018; University of Wisconsin School of Medicine and Public Health 2019. The 2019 state-specific ADI for Wisconsin was utilized.

Census tract data was used to identify the COI for each child, which measures the resources and conditions important for a child’s healthy development on a neighborhood-level. COI utilizes 29 indicators separated into three main domains of 1) education, 2) health and environment, and 3) social and economic. COI is reported by five levels that consist of very low, low, moderate, high, and very high, with very low indicating low level of opportunity and very high indicating high level of opportunity. COI is also reported by scores ranging from 1 to 100, with 1 indicating the lowest degree of opportunity and 100 indicating the highest. COI is calculated by national-, state-, and metro-based norms (Noelke et al. 2020). State-level data was utilized for this study to account for transfer patterns as Children’s Wisconsin treats patients from throughout southeast and central Wisconsin. The most recent 2.0 COI dataset was utilized.

### Statistical analysis

Comparisons between the Reinjured and Non-reinjured cohorts were performed using chi-squared tests to compare categorical variables and Wilcoxon signed-rank tests to compare continuous, skewed variables. Fisher’s exact tests were used for cell sizes ≤ 5. All missingness was presented for variables that had missing data. Both ADI and COI were compared between the Reinjured and Non-reinjured cohort to assess for differences in socioeconomic status and neighborhood-level factors. A sensitivity analysis comparing demographic and injury characteristics for 1) children with ADI and children with missing ADI, and 2) children with COI and children with missing COI was performed to explore differences in children with and without missing neighborhood-level data. Another sensitivity analysis was completed to account for possible child abuse, acknowledging the distinct nature of that population. This analysis compared Reinjured and Non-reinjured for children 5–18 years old to explore any changes in the data when children < 5 years old, whom are more likely to be injured by child abuse, were removed. The distribution of assaults was analyzed utilizing a map of Milwaukee County that depicted COI for assault-injured children by census tract. Statistical significance was set at p < 0.05. Statistical analysis was performed using RStudio© version 1.4.1717 (RStudio 2022).

### Post-hoc analyses

A post-hoc analysis evaluated COI in the total assault-injured cohort (including both reinjured and non-reinjured children), an unintentionally injured cohort, and a state-based normative cohort that was used as a proxy for a locally applicable non-injured cohort. The assault-injured cohort, which is the same cohort used in the main analysis, was obtained from ICD-10 codes. The unintentionally injured cohort was also obtained from ICD-10 codes and consisted of unique patients rather than encounters. The state-based normative cohort data was obtained from the COI database (Noelke et al. 2020). Overall COI in addition to COI by the three domains of education, health and environment, and social and economic for each of the three cohorts were assessed by both score and level.

## Results

### Overall study population

**Fig. 1:**
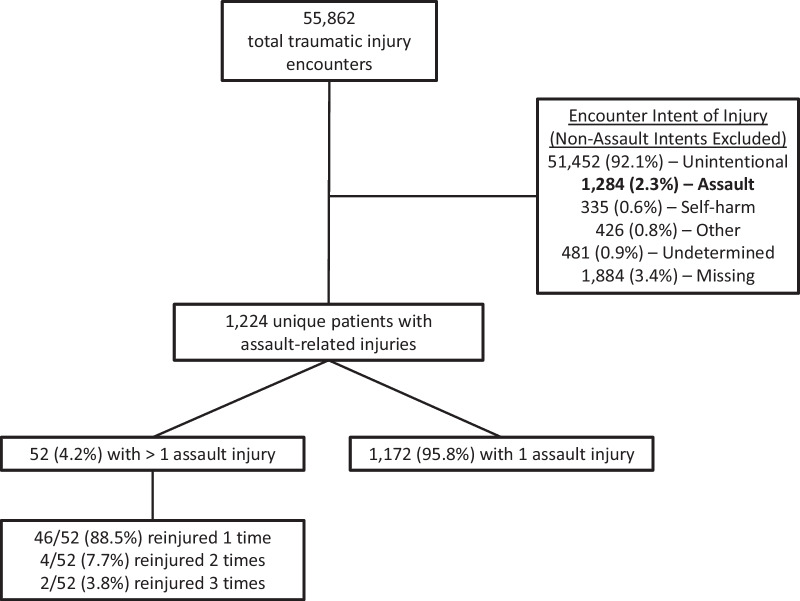
Study population for assault-injured youth

There were 55,862 traumatic injury encounters during the study period from the TR and EMR. Of those 1224 children were assault-injured, with 52 (4.2%) children identified as reinjured and 1172 (95.8%) children identified as non-reinjured (Fig. 1). The median time to reinjury was 240 days (interquartile range [IQR] 63–406 days). Of those reinjured, 46/52 (88.5%) were reinjured once, 4/52 (7.7%) were reinjured twice, and 2/52 (3.8%) were reinjured three times.

Assault-injured children had a median age of 14.0 (IQR 9.0–16.0), with 62.3% between the ages of 13–18 years old (Table 1). 18.1% of children were < 5 years old. They were predominantly Black (68.1%), non-Hispanic/Latino (84.2%), and had public insurance (82.4%). Additionally, 62.7% of assault-injured children lived in areas of middle- or high-ADI. Likewise, most children injured by assaults lived in areas of low or very low COI (64.6%). The residence of a child could not be linked to an ADI code in 15.8% (193/1224) of children. The residence of a child could not be linked to a COI code in 23.6% (289/1224) of children; 58.8% (170/289) lacked an address-matched census tract, and the remainder had a census tract that did not match to the COI database.

**Table 1:** Demographic characteristics of reinjured and non-reinjured children injured by assaults

	Total cohort N = 1224	Reinjured cohort N = 52	Non-reinjured cohort N = 1172	Significance (P value)
Median age, years (IQR)	14.0 (9.0–16.0)	15.0 (13.8–17.0)	14.0 (8.8–16.0)	< 0.001
*Age, years (N [%])*				
< 1	125 (10.2)	2 (3.9)	123 (10.5)	0.002
1–4	97 (7.9)	0 (0.0)	97 (8.3)	
5–12	239 (19.5)	5 (9.7)	234 (20.0)	
13–18	763 (62.3)	45 (86.5)	718 (61.3)	
*Sex, N (%)*				
Male	755 (61.7)	23 (44.2)	732 (62.5)	0.01
Female	469 (38.3)	29 (55.8)	440 (37.5)	
*Race, N (%)*				
White	292 (23.9)	9 (17.3)	283 (24.2)	0.14
Black	833 (68.1)	37 (71.2)	796 (67.9)	
Other	22 (1.8)	3 (5.8)	19 (1.6)	
Unknown/deceased	77 (6.3)	3 (5.8)	74 (6.3)	
*Ethnicity, N (%)*				
Hispanic/Latino	152 (12.4)	4 (7.7)	148 (12.6)	0.14
Non-Hispanic/Latino	1031 (84.2)	47 (90.4)	984 (84.0)	
Patient refused to answer	5 (0.4)	1 (1.9)	4 (0.3)	
Unknown	36 (2.9)	0 (0.0)	36 (3.1)	
*Insurance status, N (%)*				
Public	1008 (82.4)	49 (94.2)	959 (81.8)	0.09
Private	183 (15.0)	2 (3.9)	181 (15.4)	
Self-pay	28 (2.3)	1 (1.9)	27 (2.3)	
Unknown	5 (0.4)	0 (0.0)	5 (0.4)	
*Area deprivation index, N (%)*				
Low (1– < 4)	263 (21.5)	8 (15.4)	255 (21.8)	0.46
Middle (4– < 7)	672 (54.9)	32 (61.5)	640 (54.6)	
High (7–10)	96 (7.8)	2 (3.8)	94 (8.0)	
Missing	193 (15.8)	10 (19.2)	183 (15.6)	
*Child Opportunity Index, N (%)*				
Very high	7 (0.6)	0 (0.0)	7 (0.6)	0.71
High	36 (2.9)	0 (0.0)	36 (3.1)	
Moderate	102 (8.3)	5 (9.6)	97 (8.3)	
Low	372 (30.4)	20 (38.5)	352 (30.0)	
Very low	418 (34.2)	16 (30.8)	402 (34.3)	
Missing	289 (23.6)	11 (21.2)	278 (23.7)	

*IQR* interquartile range

Regarding mechanism of injury, injuries predominantly occurred by being struck (53.3%), followed by firearm injuries (11.1%) and being cut/pierced (5.7%) (Table 2). Most children had an ISS < 15 (82.3%). The concordance correlation coefficient between TR and EMR ISS was 0.57 (95% confidence interval 0.50–0.64).

**Table 2:** Injury characteristics of reinjured and non-reinjured children injured by assaults

	Total cohort N = 1224	Reinjured cohort N = 52	Non-reinjured cohort N = 1172	Significance (P value)
*Mechanism of injury, N (%)*				
Firearm	136 (11.1)	6 (11.5)	130 (11.1)	0.72
Cut/pierce	70 (5.7)	1 (1.9)	69 (5.9)	
Burn	10 (0.8)	0 (0.0)	10 (0.9)	
Struck	652 (53.3)	30 (57.7)	622 (53.1)	
Other	349 (28.5)	14 (26.9)	335 (28.6)	
Missing	7 (0.6)	1 (1.9)	6 (0.5)	
*Injury Severity Score, N (%)*				
< 15	1007 (82.3)	46 (88.5)	961 (82.0)	0.48
16–25	100 (8.2)	3 (5.8)	97 (8.3)	
> 25	117 (9.6)	3 (5.8)	114 (9.7)	

*‘Other’ includes falls (N = 8), motor vehicle crashes (N = 1), and ‘other’ mechanisms (N = 340)

In the sensitivity analysis that examined age, sex, race, ethnicity, insurance status, mechanism of injury, and ISS between children with ADI and with missing ADI in addition to children with COI and with missing COI, there were no significant differences.

### Reinjured and non-reinjured children

Children in the Reinjured cohort were more likely to be older at index injury (median age 15.0 [IQR 13.8–17.0] vs. median age 14.0 [IQR 8.8–16.0], p < 0.001) and female (55.8% vs. 37.5% female, p = 0.01; Table 1) than the Non-reinjured cohort. COI was not associated with reinjury. There were also no significant differences between race, ethnicity, insurance status, and ADI. Mechanism of injury and ISS also did not significantly differ between cohorts. A sensitivity analysis removing children most likely to be victims of child abuse was performed for children 5–18 years old and did not show any differences in findings (Additional files 2 and 3).

### Post-hoc analyses

**Fig. 2:**
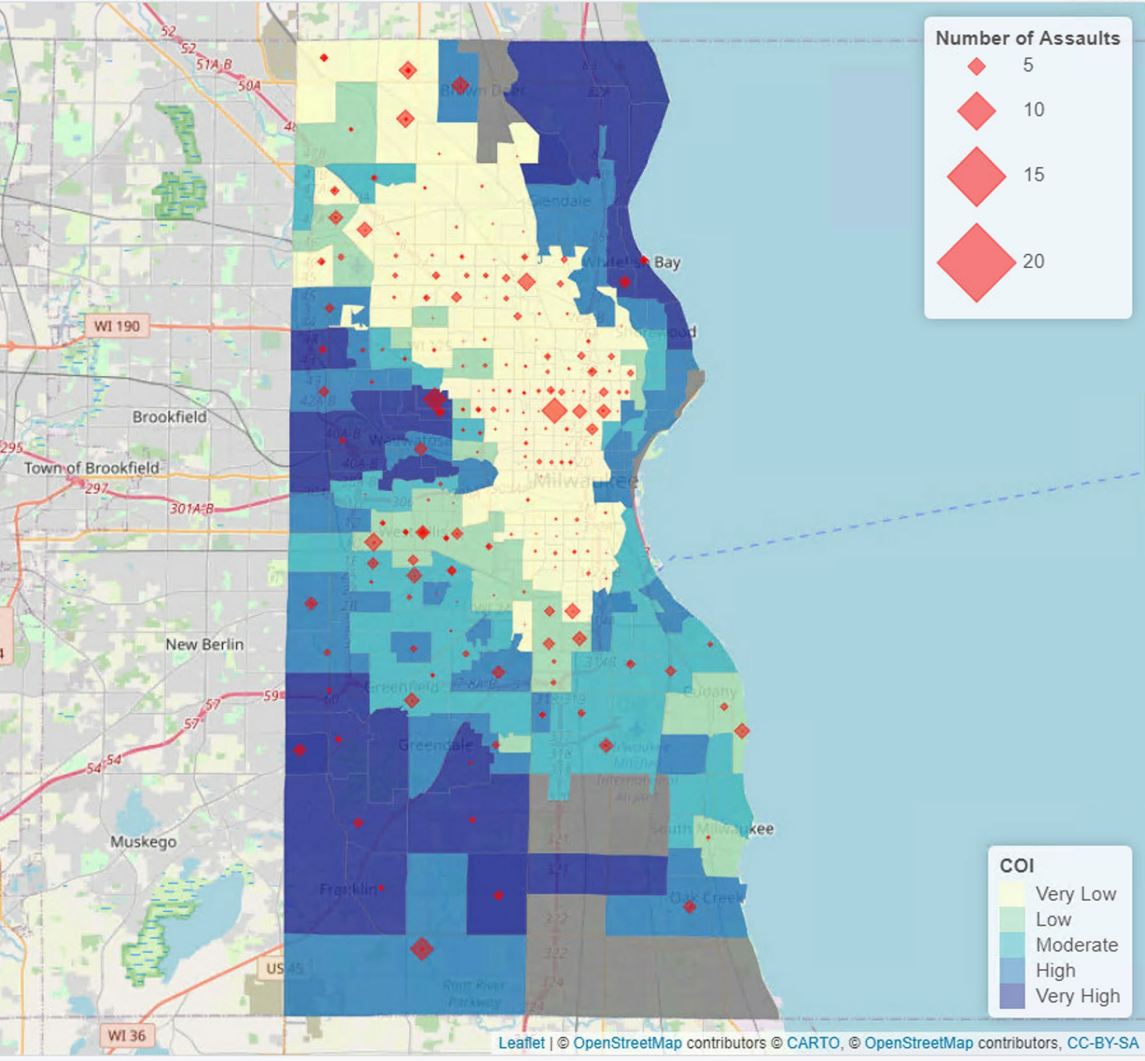
Distribution of pediatric assault-injuries in Milwaukee County in Wisconsin and the relationship to the Child Opportunity Index

Assault-injured children have significantly lower overall median COI scores and COI levels compared to the unintentionally injured and state-based normative cohorts (all p < 0.001) (Table 3). Figure 2 depicts the distribution of assault-injured children in Milwaukee County, with most children that experienced assault living in areas of low or very low COI. When evaluated by the domains of education, health and environment, and social and economic, the trend in COI scores and levels was consistent. Compared to other domains, the health and environment domain scores were the lowest among all cohorts.

**Table 3:** Comparison of Child Opportunity Index between different cohorts in Milwaukee

	Assault injured cohort* (N = 1224)	Unintentionally injured cohort* (N = 43,406)	State-based normative cohort** (N = 72,213)	Significance (P value)
*Overall Child Opportunity Index*				
Scores, Median (IQR)	9.0 (4.0–19.0)	20.0 (7.0–65.0)	48.0 (24.0–73.0)	< 0.001
Levels, N (%)				
Very high	23 (1.9)	5796 (13.4)	12,620 (17.5)	< 0.001
High	39 (3.2)	4246 (9.8)	14,378 (19.9)	
Moderate	74 (6.0)	3873 (8.9)	14,937 (20.7)	
Low	104 (8.5)	4514 (10.4)	14,940 (20.7)	
Very low	791 (64.6)	18,866 (43.5)	15,338 (21.2)	
Unknown	193 (15.8)	6111 (14.1)	0 (0.0)	
*Education domain*				
Scores, Median (IQR)	13.0 (11.0–24.0)	28.0 (12.0–69.0)	49.0 (25.0–73.0)	< 0.001
Levels, N (%)				
Very high	29 (2.4)	6332 (14.6)	13,026 (18.0)	< 0.001
High	61 (5.0)	5024 (11.6)	14,395 (19.9)	
Moderate	107 (8.7)	5810 (13.4)	14,850 (20.6)	
Low	79 (6.5)	2646 (6.1)	15,197 (21.0)	
Very low	755 (61.7)	17,483 (40.3)	14,745 (20.4)	
Unknown	193 (15.8)	6111 (14.1)	0 (0.0)	
*Health and environment domain*				
Scores, Median (IQR)	7.0 (3.0–17.5)	15.0 (5.0–39.0)	47.0 (23.0–72.0)	< 0.001
Levels, N (%)				
Very high	4 (0.3)	1925 (4.4)	12,569 (17.4)	< 0.001
High	16 (1.3)	3329 (7.7)	13,603 (18.8)	
Moderate	49 (4.0)	3842 (8.9)	14,453 (20.0)	
Low	147 (12.0)	6541 (15.1)	15,110 (20.9)	
Very low	815 (66.6)	21,658 (49.9)	16,478 (22.8)	
Unknown	193 (15.8)	6111 (14.1)	0 (0.0)	
*Social and economic domain*				
Scores, median (IQR)	8.0 (3.0–20.0)	21.0 (7.0–65.0)	48.0 (24.0–73.0)	< 0.001
Levels, N (%)				
Very high	31 (2.5)	5956 (13.7)	13,192 (18.3)	< 0.001
High	50 (4.1)	4241 (9.8)	14,048 (19.5)	
Moderate	57 (4.7)	3852 (8.9)	14,707 (20.4)	
Low	115 (9.4)	4621 (10.6)	14,779 (20.5)	
Very low	778 (63.6)	18,625 (42.9)	15,487 (21.4)	
Unknown	193 (15.8)	6111 (14.1)	0 (0.0)	

*IQR* interquartile range

*Cohorts include patients identified from ICD-10 codes used in the study

**State-based normative cohort from the Child Opportunity Index database that represents a non-injured cohort

## Discussion

This retrospective study found that COI was not associated with a decrease in reinjury among assault-injured youth, contrary to our initial hypothesis. Additionally, though reinjured children were more likely to be older at index injury and female, there were no other significant differences in other demographic and injury characteristics compared to non-reinjured children. A sensitivity analysis to account for potential child-abuse identified no differences. Additional analysis further examining COI due to similarities between the cohorts showed that all assault-injured children, irrespective of reinjury, were more likely to live in areas of lower COI compared to non-assault injured and non-injured populations.

Our findings demonstrating a lack of difference in COI between assault-related reinjured and non-reinjured children suggest these two populations are from neighborhoods with similar COI levels. In other words, if a child lives in a neighborhood where they are at risk of an initial assault-related injury, they are also at risk for reinjury. The intrinsic risk of injury and reinjury in certain neighborhoods is therefore present even before the index injury. Focusing on violence prevention and intervention efforts is critical. Project Ujima, which is a hospital-based violence intervention program in Milwaukee that utilizes community partners and home visiting services for assault-injured children contributes to addressing the perpetuation of violence (Children’s Wisconsin 2022). The lower rate of reinjury (4.2%) in our study compared to what has been identified in the literature could potentially be due to intervention programs such as Project Ujima.

Due to the role neighborhoods may have in relation to violence, identifying children most at risk is necessary for effective violence intervention and prevention against even one assault-injury from occurring. Evaluation of COI in Milwaukee’s assault-injured youth compared to other populations was important for further ascertaining these high-risk areas. Growing up in a neighborhood with less poverty and more resources has long-lasting positive effects that extend well beyond childhood (Chetty and Hendren 2018). Additionally, a study by Chetty et al. demonstrated that education and economic outcomes improve when children move to lower-poverty neighborhoods, and that the longer the duration in such neighborhoods, the better long-term success a child experiences (Chetty et al. 2016). In fact, lack of neighborhood opportunity has been associated with less years of higher levels of education, employment, and income, and worse health outcomes (Sandel et al. 2016). Furthermore, it is important to note the existence of opportunity hoarding, which is a sociological theory concerning the control of economic, social, or educational resources by certain populations that subsequently leads to the exclusion of other populations from being able to access such resources (Rury and Saatcioglu 2015). Opportunity hoarding leads to children of racial/ethnic minorities often living in neighborhoods with much lower opportunity than white children (Acevedo-Garcia et al. 2020).

Regarding demographic information, we found that reinjured children were more likely to be older at index injury and female. Mechanism of injury did not differ between populations, and reinjured children were not at higher risk of more severe injury. Most literature found that youth who are older, male, Black, with public insurance, and that live in areas of worse socioeconomic status were significantly more likely to be reinjured, though many of the studies included older populations with individuals < 25 years old (Cunningham et al. 2015; Chong et al. 2015; Cortolillo et al. 2020; Gibson et al. 2016; Tellez et al. 1995). Furthermore, most repeat injuries also occur due to firearms as opposed to blunt assaults or stab wounds (Cunningham et al. 2015; Chong et al. 2015; Davis et al. 2013).

Our finding that females were more likely to be reinjured was largely unique. A prospective study by Cunningham et al. identified female sex to be predictive of an assault-related injury (Cunningham et al. 2015); however, the majority of the literature has identified males to be at higher risk of reinjury (Chong et al. 2015; Cortolillo et al. 2020; Gibson et al. 2016; Tellez et al. 1995). A gradual increase in female violence over time, a rise in local domestic abuse, sex trafficking, and an increase in overall violence in recent years due to the COVID-19 pandemic could all be contributing factors (Fernandez 2020; Flynn-O’Brien et al. 2022; Ness 2004; Tomei 2021). Additionally, our study period included the COVID-19 pandemic, which was associated with an increase in domestic violence against women, which may have also affected female youth (United Nations Women 2021). Furthermore, males that are reinjured may be taken to an adult hospital for treatment more frequently than females, which could also explain our findings regarding females and increased risk of reinjury (Walther et al. 2014).

Generally, older age, male sex, Black race, and public insurance have been found to be associated with assault-related injuries (Chong et al. 2015; Tellez et al. 1995; Carter et al. 2017; Esparaz et al. 2021; Patel et al. 2021). These same factors also portend a higher risk of long-term mortality in those injured by assaults (Shaahinfar et al. 2018). Other aspects that are important for consideration of more risk include prior violent or weapon experience, episodes of aggression, prior substance use, mental health illness, involvement with child welfare or the juvenile court system, and family or peer conflict (Cunningham et al. 2014; Kironji et al. 2021; McCart et al. 2006; Voith et al. 2022). Most assault-injured children experienced blunt injuries by being struck, with penetrating trauma after a firearm injury being the second most common type and mechanism, respectively. Studies have shown mixed results, with some populations experiencing more firearm and/or penetrating injuries (Chong et al. 2015; Tellez et al. 1995; Davis et al. 2013), while others had findings similar to ours (Cunningham et al. 2015; Flynn-O’Brien et al. 2022).

Our study also showed that assault-injured children were more likely to live in lower socioeconomic areas. Other studies have demonstrated similar findings (Chong et al. 2015; Patel et al. 2021). The aforementioned demographic and injury characteristics are influenced by neighborhood-level factors and low levels of economic opportunity (Carter et al. 2017), all of which perpetuate a cycle of violence within the community. A study by Kersten et al. (2018) corroborated this, showing that children living in very-low opportunity neighborhoods were more than twice as likely to experience an assault injury compared to children living in very-high opportunity neighborhoods. Furthermore, it is important to note that assault injuries may occur outside the neighborhood in which a child may live. The literature is mixed as to where assault-related injuries are most likely to occur, with some finding that injuries are more likely to occur close to home while others finding that such injuries were not related to proximity to home (Haas et al. 2015; Newgard et al. 2016; Papachristos et al. 2015; Parker 2020). However, methodology and the populations evaluated varied between studies and ultimately more research is needed to assess the impact of location-based risk factors.

In our study, there were a significant number of children < 5 years old who were injured by assaults, which is usually due to child abuse (Healthychildren.org. 2022). This is a distinct mechanism with unique considerations separate from youth interpersonal violence, especially since it can also include non-physical trauma such as sexual or emotional abuse and neglect. However, we included children of all ages in the study to gain a comprehensive knowledge of child opportunity, reinjury, and which demographics of children are most at risk. Nevertheless, to gain a deeper understanding of nuances of the data, we performed a sensitivity analysis of children 5–18 years old to further examine opportunity and reinjury within the context of youth interpersonal violence. There were no differences in findings from the main analysis, highlighting that children potentially injured from child abuse did not skew the data from a cohort more likely to be injured from interpersonal violence. 

There are limitations to this study. Due to the retrospective nature of this study, there is the potential for misclassification bias, selection bias, missing data, and erroneous data. There were also limitations to the types of data that could be obtained from the TR and the EMR. However, we included both in this study to account for children that met TR criteria, in addition to ones that were seen and discharged from the Emergency Department that would have not met TR criteria. We also acknowledge the limitations imposed by using ICD-10 codes to accurately identify assault-related injury (Clery et al. 2021).

Additionally, there were limitations regarding ADI and COI due to missing data. Additionally, for COI some residences may not have linked to a census tract, and certain children may not have a linked COI in the database. However, data was missing for each of the populations evaluated so it is unlikely that this skewed our results. The lack of difference in ADI and COI between reinjured and non-reinjured children could also be due to the nature of census tract and block evaluation, which still may be too broad to account for differences in environment, resource availability, and degree of hardship. Furthermore, we were unable to identify which youth were connected to Project Ujima, which could potentially be a confounding factor for reinjury to occur or not occur depending on their involvement in the program. Regarding transfer patterns, although we are the primary referral center for the majority of moderate and severe injuries, there is the possibility that assault-injured children that are mildly injured may not be transferred to our center. And lastly, this was a cross sectional study with ‘time exposed’ being variable based on age, akin to other studies on the subject (Chong et al. 2015; Gibson et al. 2016; Tellez et al. 1995). Since reinjury was examined only during the study period and the median time to reinjury was approximately eight months after index injury, children that experienced an index assault injury at 16 or 17 years of age may have experienced a repeat injury outside of the study period. This leads to conservative estimates of reinjury. Another method would have been to establish a set follow-up period (e.g. two years) that was equal for each child (Cunningham et al. 2015); however, this would have led to missing episodes of reinjury that may have occurred after the follow-up period.

In conclusion, children who experienced reinjury were more likely to be older and female but were otherwise similar in demographic and injury characteristics to non-reinjured children. Furthermore, living in an area with more or less opportunity as measured by COI was not associated with risk of reinjury, but living in an area of lower COI was more common in assault injured youth compared to non-assault injured youth and also non-injured youth. These findings suggest that the neighborhood in which a child lives serves an instrumental role in the initiation and perpetuation of violence. Though certain demographics of children may be a focus of intervention efforts, identification of other factors on an educational, social, or environmental level that may lead to assaultive injury warrants further exploration so that successful preventative measures can be implemented. Additionally, investment in targeted interventions after index injury may help prevent reinjury.

### Supplementary Information

**Additional file 1.** Assault diagnosis ICD-10 codes.

**Additional file 2.** Sensitivity analysis of demographic characteristics of reinjured and non-reinjured children between the ages of 5-18 years old injured by assaults.

**Additional file 3.** Sensitivity analysis of injury characteristics of reinjured and non-reinjured children between the ages of 5-18 years old injured by assaults.

## Data Availability

Available in tables or as supplementary material; there are no data repositories.

## Abbreviations

COI: Child Opportunity Index
TR: Trauma registry
EMR: Electronic medical record
ICD-10: International Classification of Diseases, 10th revision
ISS: Injury Severity Score
ADI: Area Deprivation Index
