# Supplementary material for: Characteristics and neighborhood-level opportunity of assault-injured children in Milwaukee
Source: Inj Epidemiol. 2023 Aug 21;10(Suppl 1):43. doi: 10.1186/s40621-023-00453-6 (PMC10441698; doi:10.1186/s40621-023-00453-6)
Supplement: Supplementary file 1 — Additional file 1. Assault diagnosis ICD-10 codes. [file 40621_2023_453_MOESM1_ESM.docx]

| **Additional File 1. Assault diagnosis ICD-10 codes** | |
| --- | --- |
| **ICD-10 Codes** | **Description** |
| X92.0XX | Assault by drowning and submersion while in bathtub |
| X92.1XX | Assault by drowning and submersion while in swimming pool |
| X92.2XX | Assault by drowning and submersion after push into swimming pool |
| X92.3XX | Assault by drowning and submersion in natural water |
| X92.8XX | Other assault by drowning and submersion |
| X92.9XX | Assault by drowning and submersion, unspecified |
| X93.XXX | Assault by handgun discharge |
| X94.0XX | Assault by shotgun |
| X94.1XX | Assault by hunting rifle |
| X94.2XX | Assault by machine gun |
| X94.8XX | Assault by other larger firearm discharge |
| X94.9XX | Assault by unspecified larger firearm discharge |
| X95.01X | Assault by airgun discharge |
| X95.02X | Assault by paintball gun discharge |
| X95.09X | Assault by other gas, air or spring-operated gun |
| X95.8XX | Assault by other firearm discharge |
| X95.9XX | Assault by unspecified firearm discharge |
| X96.0XX | Assault by antipersonnel bomb |
| X96.1XX | Assault by gasoline bomb |
| X96.2XX | Assault by letter bomb |
| X96.3XX | Assault by fertilizer bomb |
| X96.4XX | Assault by pipe bomb |
| X96.8XX | Assault by other specified explosive |
| X96.9XX | Assault by unspecified explosive |
| X97.XXX | Assault by smoke, fire and flames |
| X98.0XX | Assault by steam or hot vapors |
| X98.1XX | Assault by hot tap water |
| X98.2XX | Assault by hot fluids |
| X98.3XX | Assault by hot household appliances |
| X98.8XX | Assault by other hot objects |
| X98.9XX | Assault by unspecified hot objects |
| X99.0XX | Assault by sharp glass |
| X99.1XX | Assault by knife |
| X99.2XX | Assault by sword or dagger |
| X99.8XX | Assault by other sharp object |
| X99.9XX | Assault by unspecified sharp object |
| Y00.XXX | Assault by blunt object |
| Y01.XXX | Assault by pushing from high place |
| Y02.0XX | Assault by pushing or placing victim in front of motor vehicle |
| Y02.8XX | Assault by pushing or placing victim in front of other moving object |
| Y02.1XX | Assault by pushing or placing victim in front of (subway) train |
| Y03.0XX | Assault by being hit or run over by motor vehicle |
| Y03.8XX | Other assault by crashing of motor vehicle |
| Y04.0XX | Assault by unarmed brawl or fight |
| Y04.1XX | Assault by human bite |
| Y04.2XX | Assault by strike against or bumped into by another person |
| Y04.8XX | Assault by other bodily force |
| Y07.01 | Husband, perpetrator of maltreatment and neglect |
| Y07.02 | Wife, perpetrator of maltreatment and neglect |
| Y07.03 | Male partner, perpetrator of maltreatment and neglect |
| Y07.04 | Female partner, perpetrator of maltreatment and neglect |
| Y07.11 | Biological father, perpetrator of maltreatment and neglect |
| Y07.12 | Biological mother, perpetrator of maltreatment and neglect |
| Y07.13 | Adoptive father, perpetrator of maltreatment and neglect |
| Y07.14 | Adoptive mother, perpetrator of maltreatment and neglect |
| Y07.410 | Brother, perpetrator of maltreatment and neglect |
| Y07.411 | Sister, perpetrator of maltreatment and neglect |
| Y07.420 | Foster father, perpetrator of maltreatment and neglect |
| Y07.421 | Foster mother, perpetrator of maltreatment and neglect |
| Y07.430 | Stepfather, perpetrator of maltreatment and neglect |
| Y07.432 | Male friend of parent (co-residing in household), perpetrator of maltreatment and neglect |
| Y07.433 | Stepmother, perpetrator of maltreatment and neglect |
| Y07.435 | Stepbrother, perpetrator or maltreatment and neglect |
| Y07.436 | Stepsister, perpetrator of maltreatment and neglect |
| Y07.490 | Male cousin, perpetrator of maltreatment and neglect |
| Y07.491 | Female cousin, perpetrator of maltreatment and neglect |
| Y07.499 | Other family member, perpetrator of maltreatment and neglect |
| Y07.50 | Unspecified non-family member, perpetrator of maltreatment and neglect |
| Y07.510 | At-home childcare provider, perpetrator of maltreatment and neglect |
| Y07.511 | Daycare center childcare provider, perpetrator of maltreatment and neglect |
| Y07.512 | At-home adultcare provider, perpetrator of maltreatment and neglect |
| Y07.513 | Adultcare center provider, perpetrator of maltreatment and neglect |
| Y07.519 | Unspecified daycare provider, perpetrator of maltreatment and neglect |
| Y07.521 | Mental health provider, perpetrator of maltreatment and neglect |
| Y07.528 | Other therapist or healthcare provider, perpetrator of maltreatment and neglect |
| Y07.529 | Unspecified healthcare provider, perpetrator of maltreatment and neglect |
| Y07.53 | Teacher or instructor, perpetrator of maltreatment and neglect |
| Y07.59 | Other non-family member, perpetrator of maltreatment and neglect |
| Y07.6 | Multiple perpetrators of maltreatment and neglect |
| Y07.9 | Unspecified perpetrator of maltreatment and neglect |
| Y08.01X | Assault by strike by hockey stick |
| Y08.02X | Assault by strike by baseball bat |
| Y08.09X | Assault by strike by other specified type of sport equipment |
| Y08.81X | Assault by crashing of aircraft |
| Y08.89X | Assault by other specified means |
| Y09 | Assault by unspecified means |
| Y38.0X1 | Terrorism involving explosion of marine weapons, public safety official injured |
| Y38.0X2 | Terrorism involving explosion of marine weapons, civilian injured |
| Y38.0X3 | Terrorism involving explosion of marine weapons, terrorist injured |
| Y38.1X1 | Terrorism involving destruction of aircraft, public safety official injured |
| Y38.1X2 | Terrorism involving destruction of aircraft, civilian injured |
| Y38.1X3 | Terrorism involving destruction of aircraft, terrorist injured |
| Y38.2X1 | Terrorism involving other explosions and fragments, public safety official injured |
| Y38.2X2 | Terrorism involving other explosions and fragments, civilian injured |
| Y38.2X3 | Terrorism involving other explosions and fragments, terrorist injured |
| Y38.3X1 | Terrorism involving fires, conflagration and hot substances, public safety official injured |
| Y38.3X2 | Terrorism involving fires, conflagration and hot substances, civilian injured |
| Y38.3X3 | Terrorism involving fires, conflagration and hot substances, terrorist injured |
| Y38.4X1 | Terrorism involving firearms, public safety official injured |
| Y38.4X2 | Terrorism involving firearms, civilian injured |
| Y38.4X3 | Terrorism involving firearms, terrorist injured |
| Y38.5X1 | Terrorism involving nuclear weapons, public safety official injured |
| Y38.5X2 | Terrorism involving nuclear weapons, civilian injured |
| Y38.5X3 | Terrorism involving nuclear weapons, terrorist injured |
| Y38.80X | Terrorism involving unspecified means |
| Y38.811 | Terrorism involving suicide bomber, public safety official injured |
| Y38.812 | Terrorism involving suicide bomber, civilian injured |
| Y38.891 | Terrorism involving other means, public safety official injured |
| Y38.892 | Terrorism involving other means, civilian injured |
| Y38.893 | Terrorism involving other means, terrorist injured |
| Y38.9X1 | Terrorism, secondary effects, public safety official injured |
| Y38.9X2 | Terrorism, secondary effects, civilian injured |
| T54.1X3 | Toxic effect of other corrosive organic compounds, assault |
| T54.2X3 | Toxic effect of corrosive acids and acid-like substances, assault |
| T54.3X3 | Toxic effect of corrosive alkalis and alkali-like substances, assault |
| T54.93X | Toxic effect of unspecified corrosive substance, assault |
| T63.013 | Toxic effect of rattlesnake venom, assault |
| T63.023 | Toxic effect of coral snake venom, assault |
| T63.033 | Toxic effect of taipan venom, assault |
| T63.043 | Toxic effect of cobra venom, assault |
| T63.063 | Toxic effect of venom of other North and South American snake, assault |
| T63.073 | Toxic effect of venom of other Australian snake, assault |
| T63.083 | Toxic effect of venom of other African and Asian snake, assault |
| T63.093 | Toxic effect of venom of other snake, assault |
| T63.113 | Toxic effect of venom of gila monster, assault |
| T63.123 | Toxic effect of venom of other venomous lizard, assault |
| T63.193 | Toxic effect of venom of other reptiles, assault |
| T63.2X3 | Toxic effect of venom of scorpion, assault |
| T63.303 | Toxic effect of unspecified spider venom, assault |
| T63.313 | Toxic effect of venom of black widow spider, assault |
| T63.323 | Toxic effect of venom of tarantula, assault |
| T63.333 | Toxic effect of venom of brown recluse spider, assault |
| T63.393 | Toxic effect of venom of other spider, assault |
| T63.413 | Toxic effect of venom of centipedes and venomous millipedes, assault |
| T63.423 | Toxic effect of venom of ants, assault |
| T63.433 | Toxic effect of venom of caterpillars, assault |
| T63.443 | Toxic effect of venom of bees, assault |
| T63.453 | Toxic effect of venom of hornets, assault |
| T63.463 | Toxic effect of venom of wasps, assault |
| T63.483 | Toxic effect of venom of other arthropod, assault |
| T63.513 | Toxic effect of contact with stingray, assault |
| T63.593 | Toxic effect of contact with other venomous fish, assault |
| T63.613 | Toxic effect of contact with Portuguese Man-o-war, assault |
| T63.623 | Toxic effect of contact with other jellyfish, assault |
| T63.633 | Toxic effect of contact with sea anemone, assault |
| T63.693 | Toxic effect of contact with other venomous marine animals, assault |
| T63.713 | Toxic effect of contact with venomous marine plant, assault |
| T63.793 | Toxic effect of contact with other venomous plant, assault |
| T63.813 | Toxic effect of contact with venomous frog, assault |
| T63.823 | Toxic effect of contact with venomous toad, assault |
| T63.833 | Toxic effect of contact with other venomous amphibian, assault |
| T63.893 | Toxic effect of contact with other venomous animals, assault |
| T63.93X | Toxic effect of contact with unspecified venomous animal, assault |
| T65.823 | Toxic effect of harmful algae and algae toxins, assault |
| T71.113 | Asphyxiation due to smothering under pillow, assault |
| T71.123 | Asphyxiation due to plastic bag, assault |
| T71.133 | Asphyxiation due to being trapped in bed linens, assault |
| T71.143 | Asphyxiation due to smothering under another person's body (in bed), assault |
| T71.153 | Asphyxiation due to smothering in furniture, assault |
| T71.163 | Asphyxiation due to hanging, assault |
| T71.193 | Asphyxiation due to mechanical threat to breathing due to other causes, assault |
| T71.223 | Asphyxiation due to being trapped in a car trunk, assault |
| T71.233 | Asphyxiation due to being trapped in a (discarded) refrigerator, assault |
| T74.01 | Adult neglect or abandonment, confirmed |
| T74.02 | Child neglect or abandonment, confirmed |
| T74.11 | Adult physical abuse, confirmed |
| T74.12 | Child physical abuse, confirmed |
| T74.21 | Adult sexual abuse, confirmed |
| T74.22 | Child sexual abuse, confirmed |
| T74.31 | Adult psychological abuse, confirmed |
| T74.32 | Child psychological abuse, confirmed |
| T74.4 | Shaken infant syndrome |
| T74.91 | Unspecified adult maltreatment, confirmed |
| T74.92 | Unspecified child maltreatment, confirmed |
| T76.01 | Adult neglect or abandonment, suspected |
| T76.02 | Child neglect or abandonment, suspected |
| T76.11 | Adult physical abuse, suspected |
| T76.12 | Child physical abuse, suspected |
| T76.21 | Adult sexual abuse, suspected |
| T76.22 | Child sexual abuse, suspected |
| T76.31 | Adult psychological abuse, suspected |
| T76.32 | Child psychological abuse, suspected |
| T76.91 | Unspecified adult maltreatment, suspected |
| T76.92 | Unspecified child maltreatment, suspected |
