# Supplementary material for: Characteristics and neighborhood-level opportunity of assault-injured children in Milwaukee
Source: Inj Epidemiol. 2023 Aug 21;10(Suppl 1):43. doi: 10.1186/s40621-023-00453-6 (PMC10441698; doi:10.1186/s40621-023-00453-6)
Supplement: Supplementary file 2 — Additional file 2. Sensitivity analysis of demographic characteristics of reinjured and non-reinjured children between the ages of 5-18 years old injured by assaults. [file 40621_2023_453_MOESM2_ESM.docx]

| **Additional File 2. Sensitivity analysis of demographic characteristics of reinjured and non-reinjured children between the ages of 5-18 years old injured by assaults** | | | | |
| --- | --- | --- | --- | --- |
|  | **Total**  **Cohort**  **N=1002** | **Reinjured**  **Cohort**  **N=50** | **Non-Reinjured**  **Cohort**  **N=952** | **Significance**  **(P-value)** |
| **Median Age, years (IQR)** | 15.0  (13.0-16.0) | 15.0  (14.0-17.0) | 15.0  (13.0-16.0) | 0.03 |
|  |  |  |  |  |
| **Age, years (N [%])** |  |  |  |  |
| 5-12 | 239 (23.9) | 5 (10.0) | 234 (24.6) | 0.02 |
| 13-18 | 763 (76.2) | 45 (90.0) | 718 (75.4) |  |
| **Sex, N (%)** |  |  |  |  |
| Male | 628 (62.7) | 21 (42.0) | 607 (63.8) | 0.003 |
| Female | 374 (37.3) | 29 (58.0) | 345 (36.2) |  |
| **Race, N (%)** |  |  |  |  |
| White | 187 (18.7) | 9 (18.0) | 178 (18.7) | 0.13 |
| Black | 736 (73.5) | 36 (72.0) | 700 (73.5) |  |
| Other | 16 (1.6) | 3 (6.0) | 13 (1.4) |  |
| Unknown/Deceased | 63 (6.3) | 2 (4.0) | 61 (6.4) |  |
| **Ethnicity, N (%)** |  |  |  |  |
| Hispanic/Latino | 124 (12.4) | 3 (6.0) | 121 (12.7) | 0.08 |
| Non-Hispanic/Latino | 850 (84.8) | 46 (92.0) | 804 (84.5) |  |
| Patient Refused to Answer | 3 (0.3) | 1 (2.0) | 2 (0.2) |  |
| Unknown | 25 (2.5) | 0 (0.0) | 25 (2.6) |  |
| **Insurance Status, N (%)** |  |  |  |  |
| Public | 822 (82.0) | 47 (94.0) | 775 (81.4) | 0.10 |
| Private | 149 (14.9) | 2 (4.0) | 147 (15.4) |  |
| Self-Pay | 27 (2.7) | 1 (2.0) | 26 (2.7) |  |
| Unknown | 4 (0.4) | 0 (0.0) | 4 (0.4) |  |
| **Area Deprivation Index, N (%)** |  |  |  |  |
| Low (1-<4) | 180 (18.0) | 8 (16.0) | 172 (18.1) | 0.57 |
| Middle (4-<7) | 581 (58.0) | 30 (60.0) | 551 (57.9) |  |
| High (7-10) | 87 (8.7) | 2 (4.0) | 85 (8.9) |  |
| Missing | 154 (15.4) | 10 (20.0) | 144 (15.1) |  |
| **Child Opportunity Index, N (%)** |  |  |  |  |
| Very High | 7 (0.7) | 0 (0.0) | 7 (0.7) | 0.84 |
| High | 25 (2.5) | 0 (0.0) | 25 (2.6) |  |
| Moderate | 83 (8.3) | 5 (10.0) | 78 (8.2) |  |
| Low | 326 (32.5) | 18 (36.0) | 308 (32.4) |  |
| Very Low | 368 (36.7) | 16 (32.0) | 352 (37.0) |  |
| Missing | 193 (19.3) | 11 (22.0) | 182 (19.1) |  |
