# Supplementary material for: Characteristics and neighborhood-level opportunity of assault-injured children in Milwaukee
Source: Inj Epidemiol. 2023 Aug 21;10(Suppl 1):43. doi: 10.1186/s40621-023-00453-6 (PMC10441698; doi:10.1186/s40621-023-00453-6)
Supplement: Supplementary file 3 — Additional file 3. Sensitivity analysis of injury characteristics of reinjured and non-reinjured children between the ages of 5-18 years old injured by assaults. [file 40621_2023_453_MOESM3_ESM.docx]

| **Additional File 3. Sensitivity analysis of injury characteristics of reinjured and non-reinjured children between the ages of 5-18 years old injured by assaults** | | | | |
| --- | --- | --- | --- | --- |
|  | **Total**  **Cohort**  **N=1002** | **Reinjured**  **Cohort**  **N=50** | **Non-Reinjured**  **Cohort**  **N=952** | **Significance**  **(P-value)** |
| **Mechanism of Injury, N (%)** |  |  |  |  |
| Firearm | 129 (12.9) | 6 (12.0) | 123 (12.9) | 0.30 |
| Cut/Pierce | 68 (6.8) | 1 (2.0) | 67 (7.0) |  |
| Burn | 3 (0.3) | 0 (0.0) | 3 (0.3) |  |
| Struck | 640 (63.9) | 30 (60.0) | 610 (64.1) |  |
| Other* | 156 (15.6) | 12 (24.0) | 144 (15.1) |  |
| Missing | 6 (0.6) | 1 (2.0) | 5 (0.5) |  |
| **Injury Severity Score, N (%)** |  |  |  |  |
| <15 | 894 (89.2) | 45 (90.0) | 849 (89.2) | 0.75 |
| 16-25 | 61 (6.1) | 2 (4.0) | 59 (6.2) |  |
| >25 | 47 (4.7) | 3 (6.0) | 44 (4.6) |  |
| *’Other’ includes falls (N=4), motor vehicle crashes (N=1), and ‘other’ mechanisms (N=151) | | | | |
